# Re-utilization of a transcription factor

**DOI:** 10.7554/eLife.21522

**Published:** 2016-10-14

**Authors:** Filipe Pinto-Teixeira, Claude Desplan

**Affiliations:** 1 Department of Biology, New York University, New York, United Statesfilipepts@gmail.com; 1 Department of Biology, New York University, New York, United Statescd38@nyu.edu; 2 Center for Genomics and Systems Biology, New York University Abu Dhabi, Abu Dhabi, United Arab Emirates; 2 Center for Genomics and Systems Biology, New York University Abu Dhabi, Abu Dhabi, United Arab Emirates

**Keywords:** cell fate specification, feedforward regulatory pathways, developmental neuroscience, *D. melanogaster*

## Abstract

The temporal transcription factor Krüppel has a dual role in the development of neurons.

**Related research article** Stratmann J, Gabilondo H, Benito-Sipos J, Thor S. 2016. Neuronal cell fate diversification controlled by sub-temporal action of *Kruppel*. *eLife* **5**:e19311. doi: 10.7554/eLife.19311

A neuronal stem cell is a cell that divides to produce another neuronal stem cell plus a cell that can go on to become any one of the different types of neurons or glial cells that are found in the nervous system. Understanding how this happens is a major challenge in developmental neuroscience. In both vertebrates and invertebrates, the capacity of neuronal stem cells to produce specific cell types is determined by a combination of spatial patterning factors, essentially determined by their birth location, and temporal patterning, in which transcription factors that are expressed sequentially in the neuronal stem cells determine the kind of cells that are produced during a given time window (reviewed in Kohwi and Doe, 2013). Now, in eLife, Johannes Stratmann, Hugo Gabilondo, Jonathan Benito-Sipos and Stefan Thor – who are based at Linköping University and Universidad Autónoma de Madrid – report that Krüppel, a transcription factor that is involved in temporal patterning, has a bigger role than was previously realized (Stratmann et al., 2016).

In *Drosophila*, neuronal stem cells, known as neuroblasts, in the ventral nerve cord (Isshiki et al., 2001), the central brain (Bayraktar and Doe, 2013) and the optic lobes (Li et al., 2013) express a series of different temporal transcription factors as they divide, and this temporal patterning dictates the identity of the neurons that are produced over time. In the ventral nerve cord, Hunchback is expressed first, followed by Krüppel, Pdm, Castor and Grainyhead. Moreover, during the time window in which a given transcription factor is expressed, two or more different types of neurons can be produced. For example, at the end of the Castor window, a neuroblast called NB5-6 divides four times to sequentially produce four different types of neurons: Tv1, Tv2, Tv3 and Tv4 neurons.

The identity of each neuron formed during the Castor window is established by a complex web of transcriptional cascades that depend on Castor and other transcription factors (see Figure 1; Baumgardt et al., 2009; Baumgardt et al., 2007; Benito-Sipos et al., 2011). However, many of the details of the mechanisms that switch these cascades on and off at different times are unknown. Stratmann et al. shed light on the subject by looking at the role of Krüppel (Kr). The neuroblast being studied will already have passed through the Kr temporal window, but this transcription factor is expressed again in the neurons that are produced during the Castor window.

**Figure 1.**
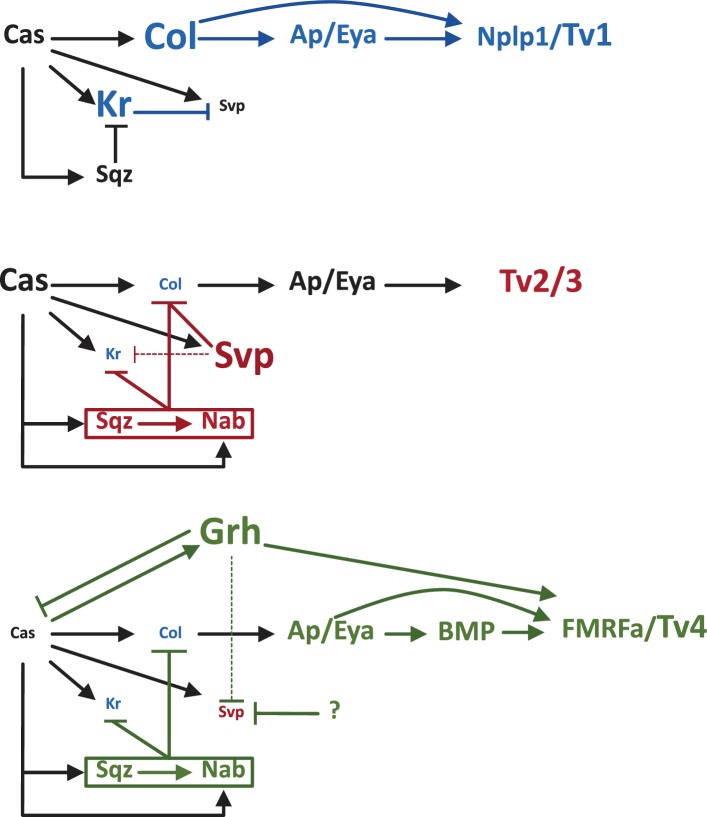
Establishing four different types of neurons in the Castor window. Castor (Cas) is a temporal transcription factor expressed in NB5-6 neuroblasts. It activates the transcription factor Collier (Col), which then promotes the expression of Apterous and Eyes absent (Ap/Eya). In Tv1, Col and Ap/Eya form a feedforward loop to activate the neuropeptide called Nlpl1. This results in the neuroblast producing a Tv1 neuron (top). In Tv2/3 neurons, Cas activates a feedforward loop consisting of Squeeze (Sqz), Nab and Seven up (Svp) that inhibits Col, blocking the feedforward loop that leads to Nplp1 expression and allowing the establishment of the Tv2/3 identity (middle). In Tv4, Cas promotes the gradual expression of the next temporal transcription factor, Grainyhead (Grh), which inhibits Cas and Svp (thus blocking Tv2/Tv3 specification), while allowing the formation of a feedforward loop involving Ap/Eya and BMP that promotes the expression of the neuropeptide FMRFa. The neuroblast now produces a Tv4 neuron. Stratmann et al. explored the role of another temporal transcription factor, Krüppel (Kr), during the Cas window. They found that, at first, Kr inhibits Svp: this allows the establishment of the Col>Ap/Eya>Nplp1 feedforward loop that leads to the formation of Tv1 neurons (top; key players shown in blue). Later in the window, the Cas>Sqz>Nab feedforward loop inhibits both Kr and Col, as does Svp, which allows Tv2 and Tv3 neurons to form (middle; key players shown in red). Later in the window, a putative factor helps Grh to repress Svp, Kr remains inhibited by Sqz and Nab, and Grh promotes the expression of FMRFa that specifies Tv4 (bottom; key players shown in green). BMP: bone morphogenetic protein.

The default fate for neuroblasts at the end of the Castor window is to produce Tv2/3 neurons. However, Stratmann et al. show that Castor first activates Kr expression specifically in Tv1 neurons, which in turn inhibits expression of the nuclear hormone receptor Seven up (Svp). This receptor normally inhibits the expression of a transcription factor called Collier: however, the inhibition of Svp allows for the establishment of a feedforward loop downstream of Collier that leads to the expression of the neuropeptide Nplp1 and the establishment of Tv1 neuron identity (Figure 1).

As the neuroblast divides to produce Tv2 and Tv3, Svp and two other proteins – Squeeze (Sqz) and Nab – are expressed: this all leads to the inhibition of Kr and the repression of Collier. As a consequence the Nplp1 feedforward loop is inhibited and the default Ap/Eya identity in Tv2/3 is established. In the last division, as the neuroblasts gets ready to switch to the next temporal window, the increased expression of Grainyhead (the next temporal transcription factor in the series) has two effects: it promotes the expression of a neuropeptide called FMRFa, and it helps to inhibit Svp expression. The end result is to prevent the establishment of the generic Tv2/3 fate to promote the Tv4 identity. However, Grainyhead cannot inhibit Svp on its own, so another factor must also be expressed during this period to help it repress Svp (Figure 1).

The implications of these observations extend well beyond the details of the NB5-6 lineage and raise additional questions: How is Kr and Sqz expression scheduled? And how is Nab expression delayed to only be present in Tv1? Such delay could be explained by the formation of a feedforward loop in which Castor and Sqz combine to promote Nab expression (Baumgardt et al., 2009). The fact that Cas is only expressed at relatively low levels in Tv1 could also explain the absence of Nab expression in this neuron. Understanding such timing mechanisms will define the logic of this gene network, from the early expression of spatial and temporal transcription factors in the neuroblasts to the expression of the terminal differentiation genes that assign unique identities to neurons.

Many of the factors that are active during the Castor window are also active during other windows. As we have seen, Krüppel is active in neuroblasts during the Kr window and is active again in Tv1 neurons produced during the Castor window. Sub-temporal factors can also have multiple roles: Svp, for example, is expressed early in neuroblasts to regulate the transition from the Hunchback window to the Krüppel window (Kanai et al., 2005), and is also expressed in Tv neurons produced during the Castor window. This raises the question of how common such dual roles are among temporal genes.

The expression of Collier requires the input of several spatial transcription factors (Gabilondo et al., 2016; Karlsson et al., 2010), but this is not the case for the temporal factors Castor and Grainyhead and for the sub-temporal factors (Kr, Sqz, Nab and Svp) downstream of Castor. This suggests that the activation of the feedforward loops, but not of temporal factors, can depend on the activity of spatial factors expressed earlier during neuroblast formation. It will be interesting to test how the earlier expression of spatial factors modulates the DNA landscape of neuroblasts, and their progeny neurons, to control their competence to respond to the activity of temporal and sub-temporal transcription factors. If we can find the answers to such fundamental questions, it will improve our ability to produce specific neurons by controlling the fate of neural stem cells.
